# Prebiotic Soup Components Trapped in Montmorillonite Nanoclay Form New Molecules: Car-Parrinello Ab Initio Simulations

**DOI:** 10.3390/life9020046

**Published:** 2019-06-04

**Authors:** Juan Francisco Carrascoza Mayén, Jakub Rydzewski, Natalia Szostak, Jacek Blazewicz, Wieslaw Nowak

**Affiliations:** 1 Institute of Computer Science, Poznan University of Technology, 60-965 Poznan, Poland; Francisco.Carrascoza@cs.put.poznan.pl (J.F.C.M.); Natalia.Szostak@cs.put.poznan.pl (N.S.); jblazewicz@cs.put.poznan.pl (J.B.); 2 European Center for Bioinformatics and Genomics, Poznan University of Technology, 60-965 Poznan, Poland; 3 Institute of Physics, Faculty of Physics, Astronomy and Informatics, N. Copernicus University, 87-100 Torun, Poland; jr@fizyka.umk.pl; 4 Institute of Bioorganic Chemistry, Poznan Academy of Sciences, 61-704 Poznan, Poland

**Keywords:** ab initio molecular dynamics, catalysis, prebiotic soup, origins of life

## Abstract

The catalytic effects of complex minerals or meteorites are often mentioned as important factors for the origins of life. To assess the possible role of nanoconfinement within a catalyst consisting of montmorillonite (MMT) and the impact of local electric field on the formation efficiency of the simple hypothetical precursors of nucleic acid bases or amino acids, we performed ab initio Car–Parrinello molecular dynamics simulations. We prepared four condensed-phase systems corresponding to previously suggested prototypes of a primordial soup. We monitored possible chemical reactions occurring within gas-like bulk and MMT-confined four simulation boxes on a 20-ps time scale at 1 atm and 300 K, 400 K, and 600 K. Elevated temperatures did not affect the reactivity of the elementary components of the gas-like boxes considerably; however, the presence of the MMT nanoclay substantially increased the formation probability of new molecules. Approximately 20 different new compounds were found in boxes containing carbon monoxide or formaldehyde molecules. This observation and an analysis of the atom–atom radial distribution functions indicated that the presence of Ca^2+^ ions at the surface of the internal MMT cavities may be an important factor in the initial steps of the formation of complex molecules at the early stages of the Earth’s history.

## 1. Introduction

The origins of life theories are based on hypothetical chemical scenarios that lead to the formation of biomolecules, starting with substances that could be found in a given proto-earth-like system [1,2,3]. These models share the assumption that there should be a way to explain the synthesis of complex biomolecules, starting from simpler molecular elements, and the notion that such a construction should happen in a scaled manner [4,5,6]. Despite still debated particular conditions present in the early Earth [7], formation of building blocks of life was possibly facilitated by appropriate physical factors such as reducing atmosphere, strong electric field, UV radiation, mineral catalytic surfaces, cometary impact, or high temperature [8]. Thus, most of those theories meet at a point where simple substances, such as ammonia, carbon monoxide/dioxide, molecular oxygen, and water, form a molecular intermediate prior to the formation of nucleotides or amino acids [9]. Studies of these elementary reactions and physical factors leading to more complex precursors of biosystems are important in understanding origins of life.

Numerous scenarios of prebiotic chemistry have been proposed so far, and many reviews on this topic have been published [6,9,10,11,12,13,14]. Probably the soundest test complementing such hypotheses was the Miller–Urey experiment [15]. In this experiment, for the first time, a series of biomolecular elementary building blocks, such as glycine and formamide, was obtained from a strongly reducing mixture of simple gases, such as methane, ammonia, hydrogen, and water. The substrates were sealed in a vessel in which an electric discharge was applied for a prolonged period of time. After the Miller–Urey test, similar experiments were performed [1,2,16] showing richer chemistry, and even formation of nucleobases [17].

Among other factors, the catalytic role of solid surfaces has been widely studied in the context of the origins of life. In this paper, we ask what may be the role of the nanoconfinement of simple molecules in montmorillonite clay in facilitating formation of the biomolecule precursors. We tackle this question using ab initio simulations which are able to indicate possible formation of new chemical species. As a part of an extended introduction, we give below selected accounts on some experimentally supported hypotheses, especially those restoring to catalytic interactions with solid surfaces and/or nanometric size compartments. More details may be found in Reference [18].

### 1.1. Possible Origins of Life Scenarios—A Short Review

In XXth century, an iron–sulphur hypothesis for the origins of life was proposed in a series of 1988–92 articles by G. Wächtershäuser, which attracted considerable attention [19,20]. In this scenario, not only high temperature (400 K) and pressure present in hydrothermal vents were important, but also the catalytic properties of iron sulfide minerals played a major role. This original hypothesis and a postulate of primitive autocatalytic metabolism were recently criticized as assumptions regarding concentrations of required reagents were unrealistic [21].

Another scenario was developed by Sutherland, Powner, Szostak, and coworkers, in which a HCN molecule was a key point to prebiotic chemistry. Notably, formaldehyde, studied here, and 2-aminooxazole were also involved in this scheme [5,10,12,22,23,24,25]. It was postulated that a linkage between amino acids, RNA, and lipids was possible through cyanosulfidic chemistry [12]. In an important paper [10], a hypothesis that all basic precursors of biological molecules were formed in cyanosulfidic reactions, partially catalyzed by minerals, was elaborated and supported by experimental data. An interesting geochemical scenario was dependent on schreibersite ((Fe,Ni)_3_P), HCN, hydrosulfide (HS^–^), copper, and ultraviolet light under postimpact conditions [3]. All of these experiments suggested that solid surfaces are potential places of catalytic reactions crucial for the origins of life.

Another group pursued a scenario based on formamide synthetic chemistry, stressing advantages and simplicity of one pot synthesis [1,26,27,28,29,30,31]. In a review by Saladino et al. [32], reactions of HCN with formamide catalyzed by various meteorites were described. The condensation of formamide on the surface of 15 minerals was analyzed as well [33]. Measuring the stability of the obtained products [34] provided useful insights into the ribonucleic acid (RNA) oligomer degradation processes. Clearly, the type of catalytic surface is important; for example, studies of iron–sulfur minerals [35] showed that basalts provide better stability for RNA oligomers than the other surfaces. 

Costanzo et al. described procedures for obtaining a set of biomolecules using UV–Vis light, electricity, heat, and high-energy proton bombardment [28]. An analysis of meteorite samples found glycine and formamide among other biomolecules on the surface [36,37].

It is worth to mention experiments that have shown reactions between formamide (NH_2_CHO) and thermal water (358 K) in the presence of meteorites, in the environment mimicking a plausible and “natural” prebiotic scenario. The results indicate that meteorites from classes: stony iron, chondrite, achondrite, effectively catalyze the synthesis of numerous organic biological compounds including carboxylic acids, nucleobases, amino acids, and sugars [38].

### 1.2. Catalytic Surfaces, Nanoconfinement, and Biogenesis

Reactions between elementary chemicals discussed in our paper (NH_3_, H_2_O, CO, formaldehyde, and HCN) have proven to be possible spontaneously [39] in condensed phase as well as in a liquid state [40]. By providing sufficient time under favorable conditions, these simple compounds are capable of forming more complex structures leading finally to nucleotides or amino acids, and polymers [41]. Nevertheless, the reactions are low-yield, and a long period of time is required to achieve substantial amounts of the products. Some natural aids can be incorporated in the physical environment, such as additional sources of energy, catalytic surfaces or nanoconfinement. These, first, increase the probability of a reaction occurrence, and second, result in a considerably faster development of various products and higher yields of reactions.

Chemical reactions in a limited space were considered in the past in the context of biogenesis. For review of this problem, we refer to a recent article by Dass et al. [42].

The properties of chemicals in a very constrained space are often much different than those in bulk. Molecular crowding increases the probability of reactive contacts, and strong electric field gradients help to polarize and direct molecules. Thus, chemistry in the compartments of nanometer dimensions is expected to have some peculiarities. One may expect that the reactivity of relatively inert molecules may be affected, to the extent that new species are created in the nanoconfinement conditions. This hypothesis is tested in this paper. Since in prebiotic times the Earth’s volcanic activity was strong, the environment might contain minerals formed from ashes and having porous structures, similar to our model of smectite MMT. Systems ready to accommodate various mixtures of elementary compounds might be quite abundant, thus we postulate that the computational studies of chemistry in nano-reactors are paramount in completing the full set of physical factors governing the formation of complex biomolecules. It is known, that theoretical studies of nanoconfinement are difficult to perform and rare. Therefore, we applied an advanced computational methodology to study the effects of trapping compounds, which were probably present in the primordial soup, on their reactivity while locked in minerals. 

MMT is a mineral, belonging to a subclass of smectites, a representative of nanoclays, named after its discovery at Montmorillon (France) in the XIXth century. It is formed by weathering of volcanic ash under poor drainage conditions or in saline environment. It has a unique structure with a layer of loosely bound positive ions (Na^+^ or Ca^2+^) located between negatively charged aluminosilicate surfaces. Because of its ability to absorb water and its catalytic properties, MMT has many applications in oil drilling industry, paper production, and dog food enrichment. MMT was considered in the past for its possible role in the origins of life. Namely, in 2003, Szostak et al. reported that the special electrical properties of MMT particles aid phospholipid vesicle formation. The formation rates were accelerated 100-fold after the addition of MMT to the solution of phospholipids [43]. These authors also hypothesized that at the same time, MMT nanopores could hold RNA molecules. Further experiments focused on MMT as a catalyst were reported by Ferris [44] , Joshi et al. [45], and Jheeta and Joshi [46]. Interestingly, clay minerals similar to MMT were found on Mars by the Opportunity probe. The current interest in the properties of this system is high mainly because of the possible carbon dioxide sequestration [47].

### 1.3. Theoretical Chemistry in Origins of Life Research

We use theoretical chemistry which is a useful and well-established approach for studies on surface catalytic effects. The adsorption of nucleobases over surface of several clays was often described the at density functional theory (DFT) level [48]. The modeling of kaolinite by the Leszczynski team [49,50] underscored the role of calcium for the adsorption of formamide over the clay’s surface. Bhushan et al. reported manganese oxides as a possible catalyst for the nucleobase synthesis [1]. 

The roles of TiO_2_ and the UV light in the formation of adenine and thymine from formamide were reported also [51,52]. More recently, Ferus et al. suggested that life started during the late heavy bombardment period of the Earth by meteorites [30]. This team modeled machinery capable of simulating shock waves in the laboratory by high-pressure effects. Their study was supported by DFT calculations and metadynamics free energy profiles calculations as well [17]. Important insights came from papers published by Goldman and coworkers, who showed (i.a.) that impact-induced shock compression of cometary ices, followed by expansion to ambient conditions, can produce complexes that resemble glycine [53,54]. DFT ab initio molecular dynamics (MD) on picosecond timescale showed that shock waves may drive the synthesis of transient C–N bonded oligomers. 

Classical “static” quantum chemical studies of reaction profile energies required “ad hoc” assumptions on reaction coordinates and fixed products. A much better (but more expensive) approach is to use ab initio MD, which does not depend on a force field. In MD, interacting substrates, usually located in a box with periodic boundary conditions, move in time, collide, and under favorable arrangements make products. 

Important contribution of ab initio MD (AIMD) to prebiotic chemistry was work by D. Marx et al. [55] The findings from this group work were summarized in an excellent review on chemistry occurring in nanoconfined water [56]. The authors modeled water in ‘moderate nanoconfinement’ between mackinawite mineral sheets. The cage consisted of two Fe_32_S_32_ parallel layers situated at the top and bottom of a supercell preserving the spacing of 5.03 Angstroms. Prebiotic peptide cycle was studied and, among others, free energy profiles of glycine reactions with small molecules were calculated using the Car–Parrinello MD method (CPMD) [57]. It was found that nanoconfined water exerts charge stabilizing effects. In comparison with ambient water some reaction barriers are strongly affected by such conditions. 

Stirling et al. (2016) used AIMD to monitor the reaction leading from NO_2_ to NH_3_ and catalyzed by the presence of iron minerals [58]. More advanced CPMD simulations were performed while studying pyrite (FeS_2_) as the catalyst [2,29,30,31,33]. Extensive CPMD modeling was also employed by Ferus et al. in a recent study of nucleic acid components formation in Miller–Urey atmosphere [17].

Several in silico studies of more elementary reactions leading to the formation of biomolecular fragments were published in the recent years. The Miller–Urey experiment was modeled by Saitta and Saija [39], who performed an illuminating theoretical analysis of elementary gases present in primordial soup, mimicking this experiment using CPMD. An external electric field was found to be a crucial factor leading to glycine formation via formamide. The same group performed ab initio MD simulations, and successfully described the reversible formation of formamide from very simple precursors NH_3_ + CO, both in gas phase and in solution [59] Notably, a new methodology for studies of elementary reactions channels was proposed in that paper. The same approach, i.e., AIMD simulations, was applied to monitor synthesis of nucleotides from nucleobases and 5-phospho-α-D-ribose-1-diphosphate [60]. Simulations showed that this reaction may happen in mildly basic pH and 400 K, a temperature postulated for prebiotic hydrothermal conditions, with a free-energy cost estimated as 1.2 and 3.3 kcal/mol for uracil and adenine, respectively.

Classical MD simulations for MMT clay were performed in the past as well (see [47,61] and the references therein), but only nonreactive force fields were applied, and MMT effects on the reactivity of biomolecule precursors have not been studied computationally yet. 

### 1.4. Our Aim

Here we test the hypothesis that MMT (or minerals with a similar composition/structure) might contribute to the formation of complex organic molecules during the prebiotic period of the Earth’s history. We do not coin any particular scenario with MMT as a key component. We do not try to reproduce full reactions paths leading from primordial soup components to known amino acids or nucleobases. Instead, we rather point out that unique catalytic properties of this nanoclay mineral might facilitate formation of variety of complex organic molecules even without extremely high temperatures, electric discharge, UV radiation, or high impact physical factors. We bring attention of the chemist community to a possible role and significance of metal ions adsorbed in minerals for origin of life studies.

In this work, we exploit the same modeling methodology as that used by Saitta and Saija [39], but we extended their approach to elucidate the hypothetical role of MMT on the path to the elementary building blocks of RNA or amino acids. We used AIMD simulations to evaluate a potential effect of calcium ions present in MMT, a catalytic role of the two-dimensional nanoconfinement of primordial soup components in the MMT nanopores, and the elevated temperature of 400 K on the probability of new compounds formation. To the best of our knowledge, this is the first CPMD study on the role of the MMT nanoconfinement in hypothetical processes related to early stages of biomolecular evolution. We have found that, in our model, MMT nanoclay alone facilitates formation of nearly 20 new organic compounds just from water, ammonia, methane, nitrogen, and carbon monoxide mixture at 20 ps time scale, even in the absence of an external electric field.

## 2. Methods

### 2.1. Systems

**Figure 1:**
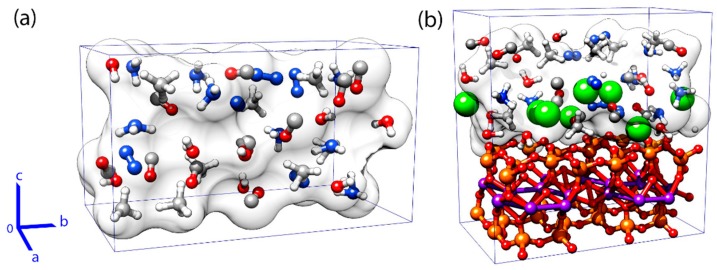
Models of g “condensed phase” (**a**) and m for “condensed phase” + montmorillonite (MMT) boxes (**b**). MMT is depicted using a “balls and sticks” representation, calcium atoms are shown as green spheres, and components of the g box are depicted using sticks and transparent surfaces. The borders of the PBC boxes and the definitions of axes are also shown.

Following the protocol presented by Saitta and Saija [39], we modeled two types of “primordial soup” systems: g and m. The g systems were virtually identical with those studied by Saitta and Saija [39] and were used here as a reference. The m systems had g mixtures confined in the slab of MMT, as shown in Figure 1 and Table 1. The g and m systems (called later “boxes”) contained components corresponding to the four stages of the glycine formation process called Miller–Strecker reaction, which were coded as follows: 0—the original Miller–Urey substrates, 1—reactants, 2—intermediates, and 3—products. The compositions of boxes were carefully selected in work [39], in order to reproduce the intermediate and end products of the Strecker reaction, and to allow each box to have a compatible number of each atom types, thus we used exactly the same systems. The boxes were electrically neutral. The preliminary initial positions of the molecules in the starting boxes were generated by PACKMOL [62] at the density of 1 g/mL. The steepest descent and simulated annealing methods were used to optimize the geometry of the initial structures. In the first step the ions’ positions were relaxed using the steepest descent algorithm, the electrons where kept on relaxation using a 0.5 ps damped electron dynamics, afterwards ions’ positions were further optimized with a damped 1 ps dynamics, and finally both, electrons and ions were relaxed once more time using the steepest descent algorithm. Then molecular dynamics was started using the Verlet algorithm for both types of particles, i.e., ions and electrons, with the increasing temperature controlled by the Nose–Hoover [63] thermostat at frequency of 13.5 Thz. Before collecting data, 2 picoseconds of equilibration in an appropriate temperature (300 K, 400 K, or 600 K) was applied in each box.

Because of the periodic boundary conditions (PBC), the mixtures in the m-type boxes were effectively confined in nanocages (~1.0 nm × 1.8 nm × 0.8 nm, see Figure 1b). The pressure in the model cavity is difficult to control and, due to the low compressibility of water, may be high. The dimensions of the boxes are presented in Table 1.

### 2.2. CPMD Simulations

CPMD simulations relay on the classical motion of heavy ions interacting with each other and experiencing a potential from fast moving electrons. In contrast to the classical MD, CPMD explicitly includes the electrons as active degrees of freedom via fictitious dynamical variables [57]. To reduce the number of electrons and computational time, atomic inner core electrons are replaced by plane–wave pseudopotentials, and electronic correlation effects are included in specially designed exchange–correlation functionals adopted from the DFT methods. Therefore, chemical bonding may be studied using CPMD.

CPMD simulations were performed using Quantum Espresso 5.3.0 [64] with the Perdew–Burke–Ernzerhov exchange and correlation functional [65] and the softcore pseudo-potentials by del Corso [66] with a kinetic energy cutoff of 35 Ry and a charge density cutoff of 280 Ry. The fictitious electronic mass was set to 500 a.u. The ion dynamics was performed in the NVT ensemble using the Verlet algorithm [67] and the Nose–Hoover [63] thermostat at a frequency of 13.5 THz. Each system was simulated at 300 K, 400 K, and 600 K, at 1 atm external pressure, for 20 ps with a time step of 0.1 fs. As already mentioned, before production runs a gradual relaxation of minimized geometry was adopted together with an increasing temperature heating phase and 2 ps equilibration runs.

The trajectories were analyzed using PLUMED 2.4 [68] and VMD [69]. A typical 20 ps CPMD run took about 12 days on a 10 processor (28 cores each) cluster at the PCSS Computing Center (Poznan, Poland). Twenty-four runs were performed.

We monitored the distributions of all of the heavy atoms forming our simple molecules from all the steps of the Miller–Urey experiment (boxes 0–3 in the g and m systems) by calculating the atom–atom radial distribution functions *g_AB_*(*r*). The radial distribution functions for atoms *B* around atoms *A* were calculated as follows

$$g_{AB}\left(r\right)=\frac{1}{4\pi \rho_{B}r^{2}}\frac{\Delta N_{A-B}}{\Delta r}$$

where *ρ_B_* is the number density of atoms B and Δ*N_A−B_* is the average number of atoms *B* lying in the region *r* to *r* + Δ*r* from a type-*A* atom.

## 3. Results and Discussion

We aimed at monitoring the chemical reactions possibly occurring in “gaseous” g-type boxes and the same molecular systems but confined in the MMT slab nanopore, i.e., m-type boxes. Further, we monitored the effects of the increasing temperature on the chemical reactivity of these mixtures as well. Twenty-four computational boxes were modeled in total (Table 1). The 20-ps timescale of the sampling ab initio mechanical dynamics is typical for such studies [39], given the high demand of computational time required for the AIMD simulations of such large systems as those studied here (126–328 atoms). The catalytic effects observed in this relatively short time window should be even more pronounced on geological timescales when not only a limited set of new compounds observed here might be formed, but formation of other complex molecules should be reasonably expected. Some of our newly formed molecules may be short lived compounds. Much longer simulations are required to sample all possible chemistry in our model systems.

Using homemade scripts and computer graphics, we searched for the formation of new chemical species. We evaluated the probabilities of reactions in each box by measuring the number of molecule–molecule close contacts, effective clashes leading to new species, and the atom–atom radial distribution functions g(r). The definition of effective clashes is given in Section 3.1.

**Figure 2:**
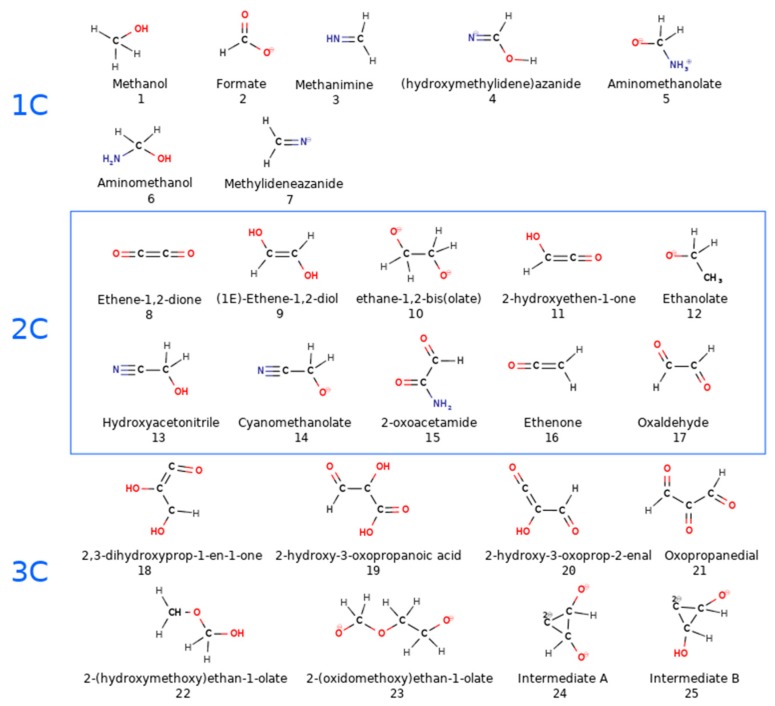
All new products noticed during 20 ps time of CPMD simulations in 24 boxes.

The dynamics of the MMT slab was also monitored by its root-mean-square deviation (RMSD), radial distribution functions, and distortions of the slab geometry by inspecting the atomic position plots for each type of metal ion. We identified the newly formed species (see Table 2 and Figure 2), and evaluated their lifetimes in the course of the 20-ps CPMD trajectories (Table 3).

### 3.1. Effects of MMT on Chemical Reactivity

We assumed that changes in the chemical reactivity or the possible catalytic effects of confinement and the presence of the MMT mineral could be monitored using the statistics of heavy atoms contacts. We defined such contact as an effective collision (clash) if two atoms A and B remained closer than a given threshold R(A–B), for a time period longer than 100 frames (725 fs). The following thresholds were adopted; R(C–C) = 1.64 Å, R(C–N) = 1.57 Å, and R(C–O) = 1.53 Å. The values were based on standard bond lengths and 0.1 Å was added to account for vibrational effects.

The results of scanning all 24 trajectories are presented in Table 2. We performed analysis of the convergence of a number of reactive clashes (where present, data not shown) and found that this number is almost constant in the 10 to 20 ps range.

First, we observed that the boxes g0 and m0 were not reactive in our simulations. Miller’s primordial soup components (water, ammonia, methane, and hydrogen) present in this box did not react spontaneously on our 20-ps timescale neither in the pure condensed phase (g) nor after the confinement and presence of MMT (m). Moderate or elevated temperatures (400 K or 600 K) did not affect this observation. This result was not surprising, as it is known that g0 shows no spontaneous reactivity and an electric field (an electric discharge) was required in the experiment to initiate the production of an amino acid with a reasonable yield. Saitta and Saija did not observe any reactivity in the identical box in their 20-ps CPMD simulations [39]. Neither the presence of the mineral confinement nor a local electric field from Ca^2+^ ions increased reactivity in this mixture. It is worth mentioning that the chemical conditions assumed in the Miller–Urey experiment are sometimes disputed, since the early Earth atmosphere had probably lower concentrations of ammonia and hydrogen [7]. The lack of reactivity observed in the CPMD modeling indicates that additional physical factors were required to trigger complex organic molecules formation.

Identical results (no reactive clashes) were observed for boxes g3 and m3. At all of the considered temperatures, the chemical composition of these boxes was constant; the boxes contained a 1:1 glycine and ammonia mixture. Interestingly, even the presence of eight Ca^2+^ ions on the surface of the MMT crystal did not activate glycine or ammonia toward making a new compound, at least within our simulation timescale. We observed numerous collisions in m3 box (Table 2) but these were transient ammonia–glycine encounters that did not result in any new stable molecules. This result is encouraging with respect to the problem of the origin of life: once formed, a simple amino acid (such as glycine here) has a good chance to remain stable even under the harsh conditions of confinement and the presence of the clay with strong local electric fields.

In contrast, boxes 1 and 2 (reactants and intermediates in model Miller’s experiment, see Table 1 for the composition) exhibited reactivity under both isolated and confined settings, leading to 25 new chemical species (see Figure 2). We divided them into group 1C (compounds **1**–**7**), 2C (**8**–**17**), and 3C+ (**18**–**25**) according to the number of carbon atoms present.

The components of g1 remained inert at all of the three temperatures; the same outcome as that reported in the Saitta and Saija simulations [39]. However, the same molecules from box 1 (water, ammonia, methane, nitrogen, and carbon monoxide) underwent frequent reactive collisions in our MMT pore model, leading to the formation of 11 different products (see Table 2 and Figure 2). There were four compounds having a new short C–C chain (ethane-1,2-dione, (E)-ethene-1,2-diol, 2-hydroxyethen-1-on, and 2-oxoacetamide; nos. **8**, **9**, **11**, and **15** in Figure 2) and four compounds (nos. **18**, **19**, **20**, and **21**) with a three-carbon atom long chain. Among them, the notable one was 2-hydroxy-3-oxopropanoic acid (no. 19), which contained a newly formed carboxylic group—a fundamental part of all of the known elementary amino acids. Further, the observed formation of 2-oxoacetamide (**15**) was considerably important, albeit it was formed only at 600 K in the m1 box.

**Figure 3:**
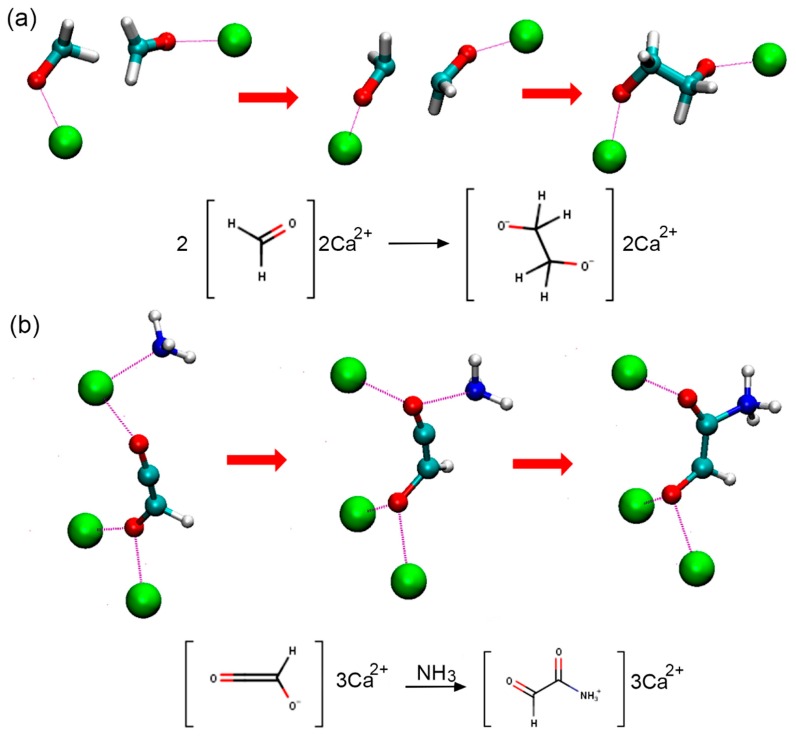
(**a**) Snapshots from a m2400 CPMD trajectory illustrating a new C–C bond formation. The formaldehyde ion is activated by Ca^2+^ (green spheres) and reacts with carbon monoxide forming oxaldehyde ion (Figure 2, **17**). (**b**) Snapshots from m1600 CPMD trajectory show a new C–N bond formation. The oxaldehyde ion (**17**) coordinated by two Ca^2+^ ions react with ammonia forming 2-oxoacamide (Figure 2, **15**).

**Figure 4:**
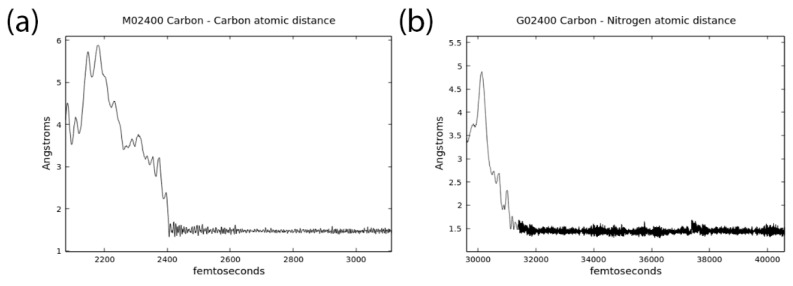
Distances between selected atoms in a fragment of CPMD trajectory showing C–C bond formation (**a**) and N–C bond formation (**b**) in m2400 box.

We found that the most numerous and diverse products were generated in the box m2400, which corresponded to the simulations at 400 K. Frequencies of reactive clashes, summarized in Table 3, illustrate this finding. Eight new compounds were observed (**1**, **5**, **6**, **7**, **10**, **14**, **23**, and **22**; Table 2 and Figure 2). There are molecules having up to six heavy atoms connected in one chain (**22** and **23**). We observed the formation of methanol (**1**). As in box 2, we initially had formaldehyde and HCN, and we observed the formation of critically important new carbon–nitrogen bonds in azaniumylmethanolate and aminomethanol (**5**, **6**). Interestingly, aminomethanol (**6**) was formed in purely g boxes g2300 and g2400, but almost all of the other complex products, except (**14**) required the presence of MMT. The snapshots of representative reactive collisions are presented in Figure 3, and the typical time evolution of the distances between the reacting fragments are shown in Figure 4.

Once the new bond is formed it typically last for many picoseconds (see Figure 4), though one should note that not all discovered species survive till the end of our 20 ps CPMD runs. Several of newly formed species thus they may have a transient nature and not necessarily lead to stable products. The formation of a new bond was clearly facilitated by the activation of polar molecules containing oxygen through interactions with the Ca^2+^ ions. In our model system, we did not introduce any additional water molecules, usually present in such a clay, except those already assigned to the reaction mixture, to mimic, or rather to enhance, the strong local electric field present on the surface (or in the pores) of the MMT mineral. These calcium ions were therefore considerably mobile and exerted strong activating catalytic effects on water, formaldehyde, and carbon monoxide. To a lesser extent, ammonia was activated by Ca^2+^. These observations were based on the visual inspection of all of the trajectories and data presented in Table 3, where statistics of the close contacts of different heavy atoms with calcium ions is presented. We observed that electronegative oxygen atoms were far often more coordinated to Ca^2+^ than neutral carbon atoms. As expected, the number of clashes increases with an increase in the temperature (Table 3).

One should note that in the regions close to the surface of minerals, say 3–5 Å, quite often strong electric fields are present. For example, in a recent paper by Laporte et al. [70], in the vicinity of a hydrated MgO surface, an electric field of 1–3 V/Å was calculated, and the electrostatic potential goes up to 5 eV. We estimated that in our MMT slab system electric field is of the same order of magnitude far from ions and surface, but the main catalytic effect comes from the presence of a very high field introduced by Ca^2+^ ions (see Supplementary Materials Figure S6).

The MMT slab remained considerably stable through all the simulations. This stability was confirmed by the low values of RMSD calculated along the 20-ps trajectories: the highest average RMSD values were calculated to be 0.38 Å, 0.57 Å, and 0.72 Å for the Si, Al, and Ca ions, respectively (see Table 4). Clearly, the Ca^2+^ ions were mobile in our models as expected; this observation was confirmed by the trace plots shown in Figure S3 (in Supplementary Materials). In real MMT clays, the alkali atoms (Ca^2+^ and Na^+^) have variable stoichiometry, are mobile, and coordinate labile water molecules [61].

Two types of reactive collision mechanisms in the m systems were qualitatively distinguished: (i) triggered by the catalytic role of calcium ions (i.e., strong local electric field) and (ii) nucleophilic bimolecular substitution in which one bond is broken and another bond is formed synchronously (a S_N_2 mechanism). In our simulations, in m type boxes, both types were represented, while in g type boxes, water-assisted polarization of ammonia and formaldehyde preceded S_N_2 type new bond formation.

It would be interesting to discriminate between possible catalytic role of confinement of small molecules in a limited space in a clay cage (m0–m3 boxes), and the role of strong local electric fields possibly exerted by Ca^2+^ ions. A more systematic computational study of this problem requires collecting extensive statistics, using other model systems and calls for a separate study.

### 3.2. Primordial Soup Ingredient Dynamics

One may expect, inspired by the observation made by Szostak group for phospholipids [43], that the MMT surface effects may lead to the preferential adsorption and ordering of components of the primordial soup. We monitored the distributions of all of the heavy atoms—C, N, and O—from basic steps of the Miller–Urey experiment (boxes 0–3 in the g and m systems) by calculating the atom–atom radial distribution functions g_AB_(r) (for definition cf. Methods).

**Figure 5:**
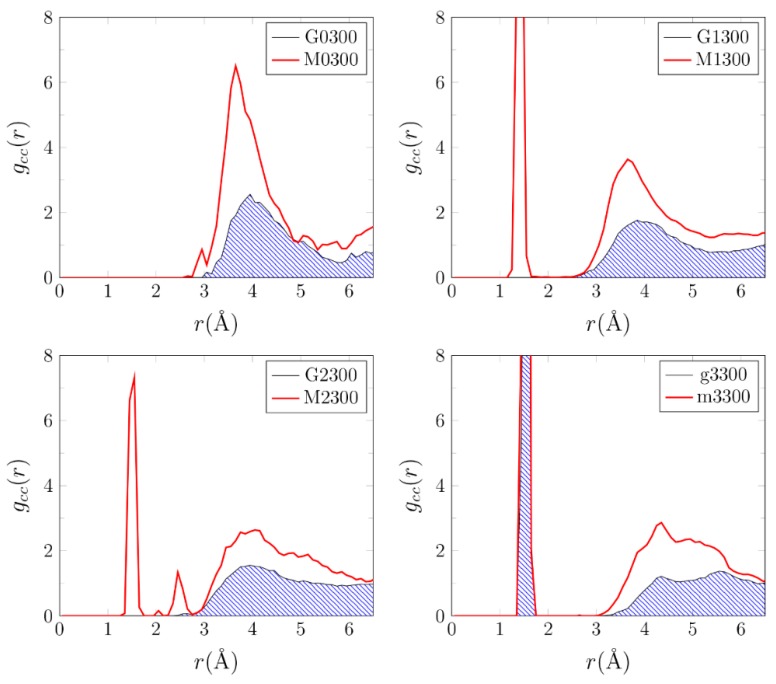
Radial carbon-carbon distance distribution functions, g_CC_(r) (in a.u.), calculated along 20-ps CPMD trajectories at 300 K.

Data are presented in Figure 5, Figures S1 and S2.

In Figure 5, we compare g_CC_(r) calculated for the g and m boxes at 300 K. The presence of MMT changed the distributions of the carbon–carbon distances. The most notable effect was a substantial increase, by a factor of 2, in the population of the carbon pairs observed at the distance of 4 Å. The narrow maxima in g_CC_(r) at 1.5–2.0 Å were attributed to the fact that the Ca^2+^ ions and the MMT surface tended to coordinate CO, formaldehyde, and glycine. The lack of such a maximum in g_CC_(r) for the g0300 box might be explained by the lower number of carbon atoms in this mixture than in the other ones (8 vs. 18) and the fact that in g0, carbon atoms were present only in CH_4_, i.e., a nonpolar molecule not coordinated by the clay ions. A similar ordering effect of MMT was also observed at 400 K and 600 K (see Figure S1). The plots of g(r) for the calcium ion–any heavy atom distances varied from box to box but depended only slightly on temperature (see Figure S2). The Ca^2+^ ions exhibited (as expected) considerable mobility as they were loosely coupled to the Si–Al mineral core (see Figure S3). In contrast, the positions of the Al and Si ions did not change considerably during the CPMD trajectory and the vibrations of the crystal were within a reasonable range (Figures S4 and S5).

The simplified model of MMT cage adopted is not perfect. Clays have variable stoichiometry, interlayer distances, defects, smaller, and variable mobile ions (Ca^2+^ and Na^+^) density. However, these theoretical data clearly showed that the nanoconfinement in MMT changed the dynamics of all of the elementary mixtures 0–3, mimicking to some extent the primordial soup. In general, carbon atoms were localized closer to each other and this effect alone increased the probability of the formation of more complex molecules. This was particularly observed in the elevated temperature simulations (m1400 and m2400). The shorter C–C distances in MMT may be only partially attributed to possible higher pressure present in these boxes. We would rather explain this reactivity by the strong polarizing effects of Ca^2+^ ions present in our model. We packed as many as eight ions in a small volume just to maintain the stoichiometry of the MMT nanoclay, and to increase the probability of reactions (if any) in our short time scale AIMD simulations.

The presence of sulfidic anions (HS^−^, HSO^3−^, and SO_3_^2−^) in certain areas of shallow water was proposed to be critical for formation of biomolecular systems [71]. Such mixtures are worth studying using the theoretical framework presented here. It is also worth to explore possible effects of internal cavity pressure and temperature-induced changes in density of the reacting mixtures neglected in our study. Reach chemistry observed upon the nanoconfinement opens also a possibility for further computational tests of alternative scenarios leading to elementary precursors of biomolecules relevant for emergence of life. Calculations of free energy profiles along reaction pathways, not only classical [72], but similar to those proposed in [17] would be also desirable but are beyond the scope of this exploratory work. Since we have found many quickly formed molecules from the intermediate Miller–Urey test boxes m1 and m2, this indicates that some complex, but not necessarily useful (“waste”), compounds might be formed in the early Earth conditions discussed here as well [73].

## 4. Conclusions

Life is based on complex molecules formed from simpler components. In the discovering of the very first steps in the origins of life, various scenarios of the formation of such elementary building blocks have to be considered. In this paper, we have addressed an intriguing question: To what extent does the confinement of the components of the hypothetical primordial soup affect the synthesis of new, more complex chemical compounds? We placed several (discussed in the literature) test mixtures in the nanopores of the MMT mineral model, frequently considered as a catalyst in the formation of biology-related compounds. Using advanced CPMD simulations, we have compared the propensity to reactivity of four standard chemical mixtures localized in a condensed phase environment (modeled by applying PBC) and an MMT nanoclay slab. The structural model of the mineral was based on crystallography data, except for the presence of a grid of eight nonhydrated Ca^2+^ ions, which were introduced to mimic the effect of a strongly localized electric field. The ions were located in typical crystallographic positions of their hydrated counterparts, and were hydrated by the water molecules present in the mixture studied. The system was therefore relatively crowded but within physical limits. The effects of nanoconfinement were dependent on a chemical composition of the prebiotic soup mixture. 

Boxes m0 and m3 remained nonreactive despite presence of the MMT model slab and Ca^2+^ ions. We found that even within a relatively short timescale of 20 ps, the MMT cavity substantially increased the reactivity of boxes 1 (water, ammonia, methane, nitrogen, and carbon monoxide) and 2 (water, ammonia, formaldehyde, and cyanide), which were composed of the intermediates of the Strecker reaction as discussed in the Miller–Urey experiment. As expected, at the elevated temperatures (400 K and, added for a reference to earlier paper, 600 K), the catalytic effectivity of MMT was higher and the largest number of more than 20 diverse products/intermediates was observed at 400 K. The elevated temperature, especially 400 K, could have been easily achieved locally in the Earth Hadean Era hydrothermal conditions. Among other species, we observed the formation of important carboxylic group and 2-oxoacetamide. Therefore, we have concluded that both the presence of Ca^2+^ and the confinement led to a higher probability of reactive collisions in some of the mixtures studied. The detailed discrimination what factor, namely Ca^2+^ ions or the nanoconfinement, plays a major role in this increased reactivity requires additional extensive and statistically sound tests. Such research requires large computational resources and was out of the scope of present study. Notably, these effects were present only if the chemical composition of the boxes was adequate; for example, for both the Miller–Urey experiment substrates (methane, hydrogen, ammonia, and water; box 0) and the products (ammonia and glycine; box 3), the MMT mineral did not exhibit any catalytic activity on 20 ps simulations time scale. Thus, our study adds new arguments supporting the popular notion that mineral surfaces and compartmentalization have to be considered as important factors in the origin of complex organic molecules. We think that such molecules may be critical for biological systems formation, both in terrestrial and extraterrestrial settings.

## Figures and Tables

**Table 1:** Systems modeled, dimensions: *a* = 10.30 Å, *b* = 17.96 Å.

Box	Temperature	No. Atoms	Chemical Species	Dimension *c* [Å]
**g0300**	300	176	32 H2O; 8 NH3; 8 CH4; 4 H2	10.43
**g0400**	400			
**g0600**	600			
**g1300**	300	126	8 H2O; 8 NH3; 8 CH4; 5 N2; 10 CO	7.41
**g1400**	400			
**g1600**	600			
**g2300**	300	126	9 H2O; 9 NH3; 9 Formaldehyde; 9 HCN	7.41
**g2400**	400			
**g2600**	600			
**g3300**	300	126	9 NH3; 9 Glycine	7.41
**g3400**	400			
**g3600**	600			
**m0300**	300	328	4 MMT; 32 H2O; 8 NH3; 8 CH4; 4 H2	18.07
**m0400**	400			
**m0600**	600			
**m1300**	300	278	4 MMT; 8 H2O; 8 NH3; 8 CH4; 5 N2; 10 CO	17.90
**m1400**	400			
**m1600**	600			
**m2300**	300	278	4 MMT; 9 H2O; 9 NH3; 9 Formaldehyde; 9 HCN	17.90
**m2400**	400			
**m2600**	600			
**m3300**	300	278	4 MMT; 9 NH3; 9 Glycine	17.90
**m3400**	400			
**m3600**	600			

**Table 2:** Number of newly formed effective atomic contacts (clashes) in each 20-ps Car–Parrinello MD method (CPMD) trajectory and a list of the observed new transient products in each box. For the chemical formulas of the products, see Figure 2.

Box	C C	C N	C O	Products
**g0300**	0	0	0	0
**g0400**	0	0	0	0
**g0600**	0	0	0	0
**g1300**	0	0	0	0
**g1400**	0	0	0	0
**g1600**	0	0	0	0
**g2300**	0	15	0	6
**g2400**	0	1	43	6
**g2600**	6	0	0	14
**g3300**	0	0	0	0
**g3400**	0	0	0	0
**g3600**	0	0	0	0
**m0300**	0	0	0	0
**m0400**	0	0	0	0
**m0600**	0	0	0	0
**m1300**	30	0	5	24, 25
**m1400**	24	0	12	8, 9, 11, 18, 19, 20, 21
**m1600**	4	0	3	9, 11, 12, 15, 18, 21
**m2300**	9	7	2	1, 3, 4, 5, 13, 22
**m2400**	17	2	9	1, 5, 6, 7, 10, 14, 22, 23
**m2600**	17	1	6	1, 2, 3, 6, 7, 10, 12, 13
**m3300**	0	7	16	0
**m3400**	0	20	15	0
**m3600**	0	7	4	0

**Table 3:** The average number of atomic Ca–X short contacts (clashes) per picosecond during 20 ps CPMD trajectories. The contact is defined as a Ca–X distance smaller than 2.5 Å.

Box	C	H	N	O
**m0300**	0	126	30	905
**m0400**	0	108	30	964
**m0600**	0	140	32	1083
**m1300**	10	41	37	649
**m1400**	10	19	56	666
**m1600**	8	47	50	882
**m2300**	3	13	70	518
**m2400**	4	28	90	603
**m2600**	9	8	181	568
**m3300**	0	1	9	825
**m3400**	0	3	30	915
**m3600**	0	4	20	985

**Table 4:** Root-mean-square deviation (RMSD) (in Å) for the MMT atoms. Data from 20-ps trajectories of m type boxes. Each analyzed trajectory contains 5000 frames.

Box	Al			Si			Ca		
	Avg.	Min.	Max.	Avg.	Min.	Max.	Avg.	Min.	Max.
**m0300**	0.17	0.03	0.24	0.18	0.04	0.24	0.63	0.03	0.86
**m0400**	0.20	0.04	0.27	0.21	0.08	0.24	0.43	0.03	0.75
**m0600**	0.28	0.06	0.39	0.29	0.06	0.39	0.62	0.05	1.00
**m1300**	0.38	0.01	0.58	0.36	0.01	0.51	0.66	0.05	1.00
**m1400**	0.21	0.07	0.34	0.25	0.04	0.46	0.51	0.04	0.82
**m1600**	0.27	0.11	0.39	0.54	0.06	1.00	0.58	0.04	0.99
**m2300**	0.16	0.04	0.38	0.46	0.03	0.55	0.66	0.05	1.00
**m2400**	0.21	0.04	0.34	0.43	0.03	0.59	0.72	0.03	1.00
**m2600**	0.28	0.04	0.45	0.57	0.04	0.73	0.71	0.04	0.99
**m3300**	0.25	0.04	0.45	0.22	0.04	0.30	0.28	0.03	1.00
**m3400**	0.18	0.05	0.50	0.25	0.05	0.42	0.36	0.03	0.52
**m3600**	0.29	0.05	0.28	0.37	0.06	0.48	0.56	0.05	1.00

Abbreviators: Avg. = average; Min. = minimum; Max. = maximum.

## Supplementary Materials

The following are available online at https://www.mdpi.com/2075-1729/9/2/46/s1, Figure S1: Radial distribution functions g(r) (in a.u.) for carbon-to-carbon distances r (in Å) at 300 K, 400 K, and 600 K in all the simulated systems, Figure S2: Radial distribution functions g(r) (in a.u.) for distances between a heavy atom and the calcium ions in all the simulated systems, Figure S3: Traces of calcium ions projected on the plane axc. Figure S4: Traces of aluminum ion positions projected on the plane axb, Figure S5: Mobility of silicon atoms represented by traces projected on the axb plane’ Figure S6: Model total electric potential (in V) calculated for selected frames from m1 and g1 CPMD trajectories.

## Abbreviations

a.u.	atomic units
Å	Angstroms
AIMD	ab initio molecular dynamics
Approx.	approximately
atm	atmospheres
CCL2	CC chemokine ligand 2
CCR2	CC chemokine receptor 2
CCR5	CC chemokine receptor 5
CPMD	Car-Parrinello molecular dynamics
DFT	density functional theory
Dimension *c*	refers to the size of the vector *c*
fs	femtoseconds
g	gases only box
g/mL	grams per milliliter
g(r)	refers to the atom-atom radial distribution function
HCN	hydrogen cyanide
i.a.	Latin meaning “among other things”
i.e.,	Latin meaning “in essence/in other words”
K	Kelvin
kcal/mol	kilocalories per mol
m	montmorillonite and gases box
MD	molecular dynamics
MMT	montmorillonite
nm	nanometer
NVT	refers to the canonical enssemble at
PBC	periodic boundary conditions
pH	potential hydrogen
ps	picoseconds
RMSD	root mean square deviation
RNA	ribonucleic acid
Ry	Rydberg atomic units
THz	terahertz
TLC	thin layer chromatography
UV	ultraviolet
UV–Vis	ultraviolet-visible
