# Supplementary material for: Prebiotic Soup Components Trapped in Montmorillonite Nanoclay Form New Molecules: Car-Parrinello Ab Initio Simulations
Source: Life (Basel). 2019 Jun 4;9(2):46. doi: 10.3390/life9020046 (PMC6617125; doi:10.3390/life9020046)
Supplement: Supplementary file 1 [file life-09-00046-s001.zip › MMT_LIFE_WNowak_SuplementaryMaterials.docx]

**Supplementary Materials**

**
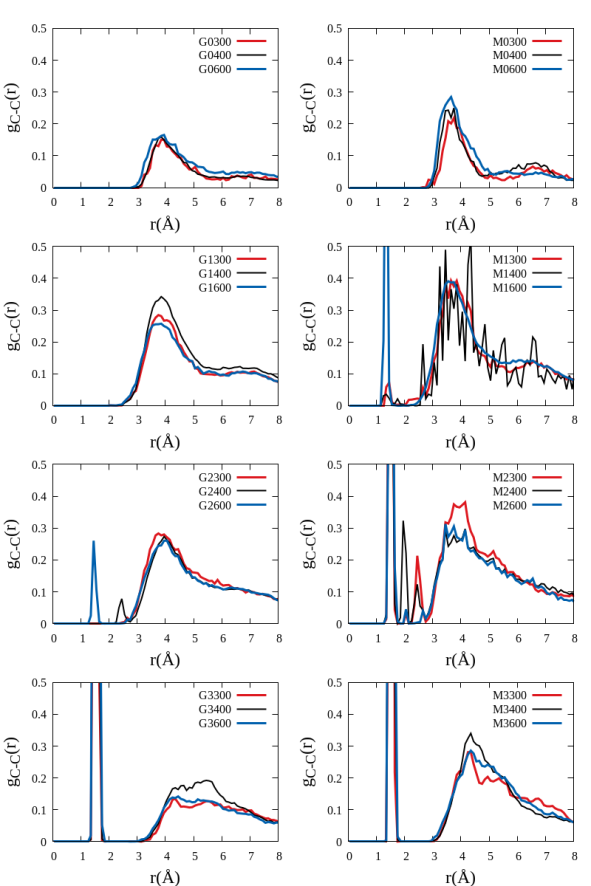
**

Figure S1: Radial distribution functions g(r) (in a.u.) for carbon-to-carbon distances r (in Å) at 300 K, 400 K, and 600 K in all the simulated systems.


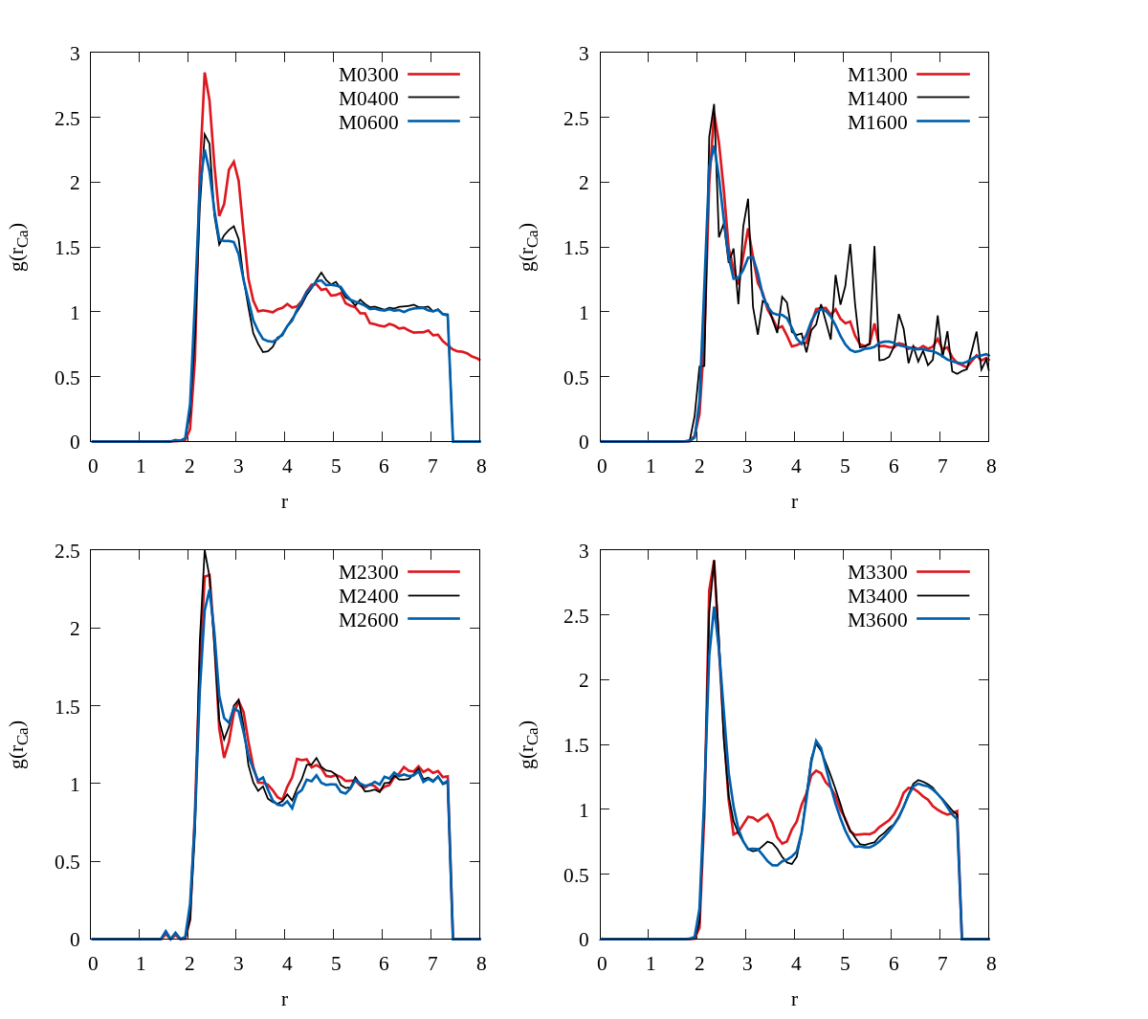


Figure S2: Radial distribution functions g(r) (in a.u.) for distances between a heavy atom and the calcium ions in all the simulated systems.


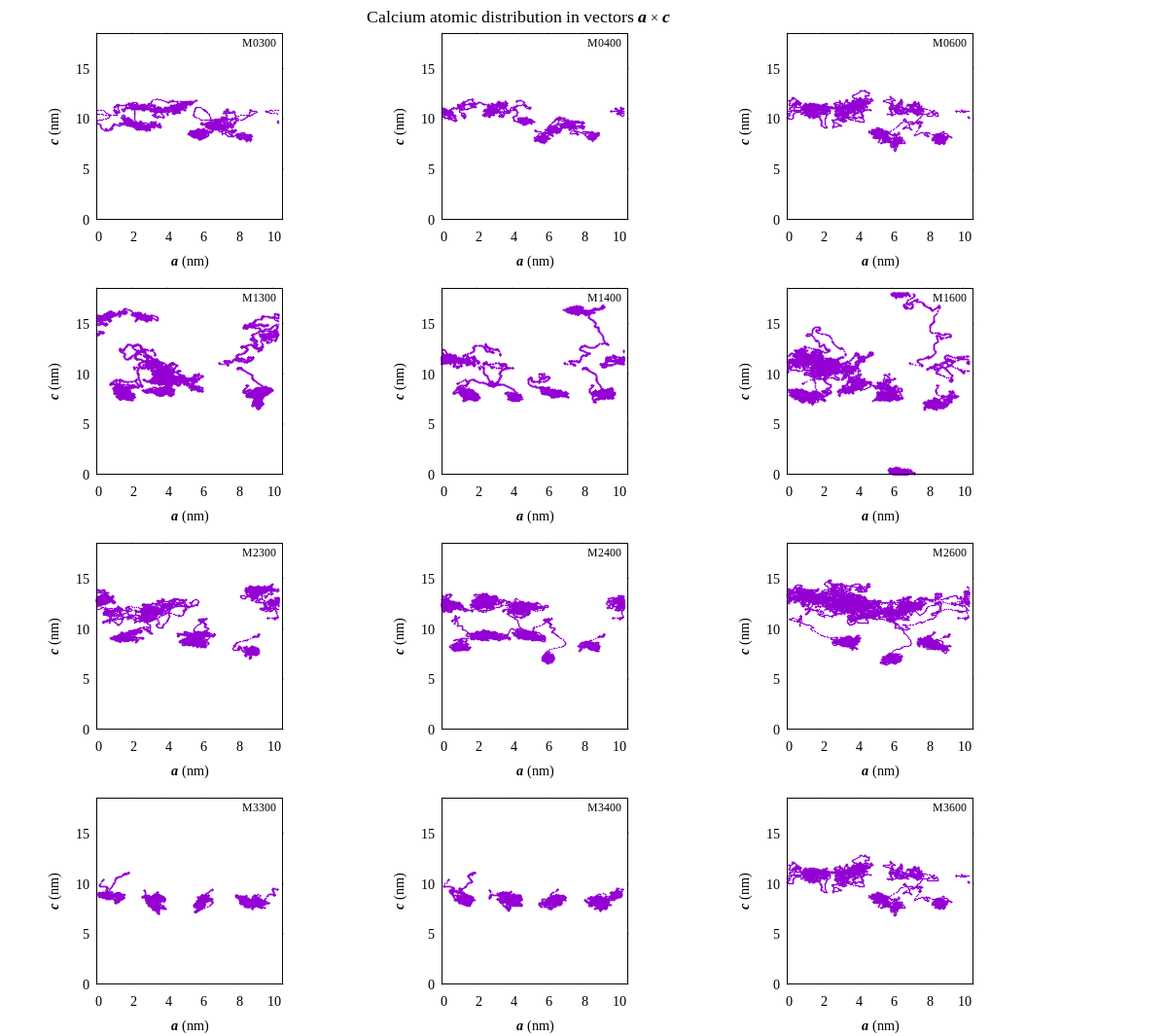


Figure S3: Traces of calcium ions projected on the plane ***a****x****c*** show the high mobility of Ca^2+^.


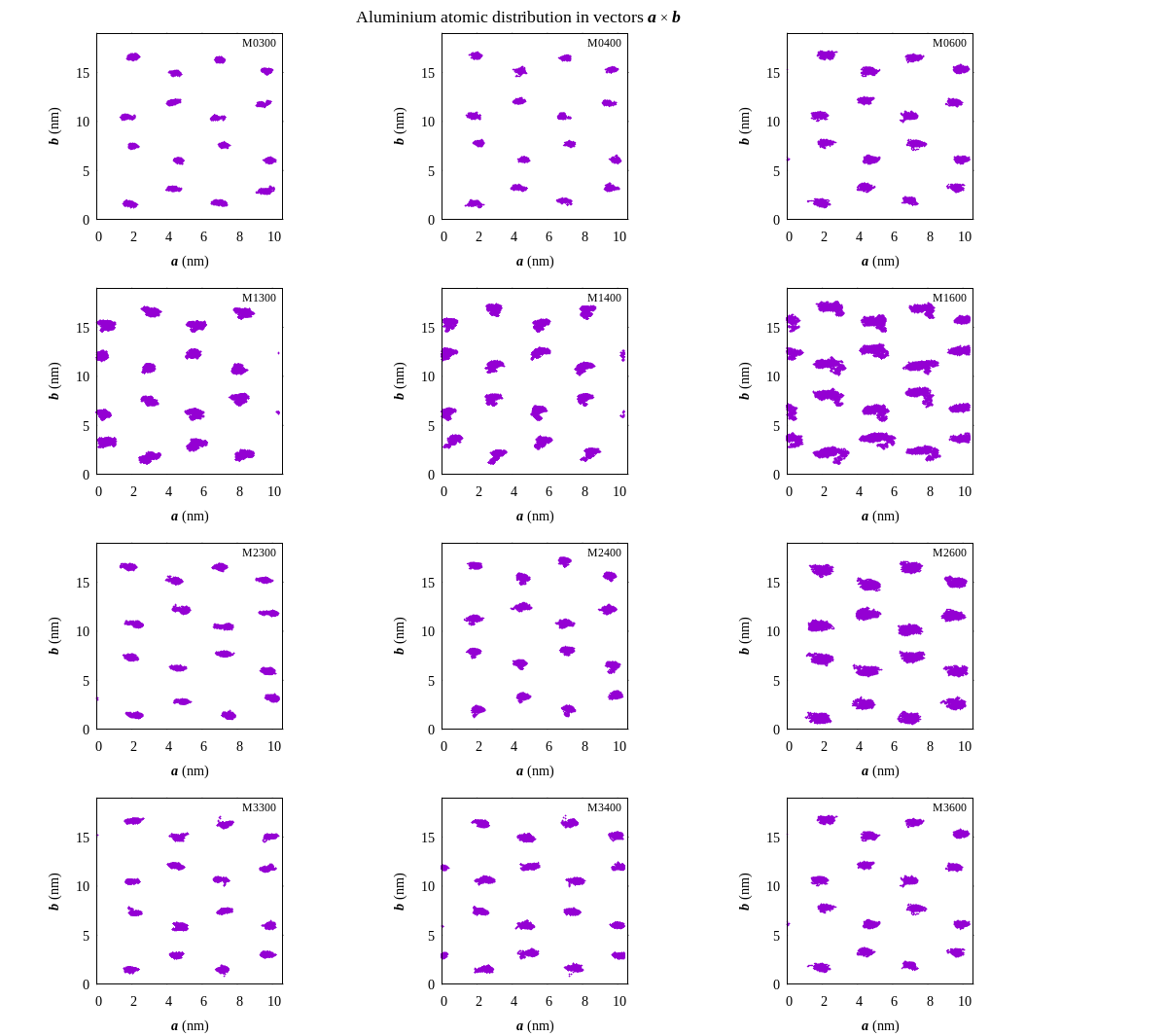


Figure S4: Traces of aluminum ion positions projected on the plane ***a****x****b*** indicate their low mobility.


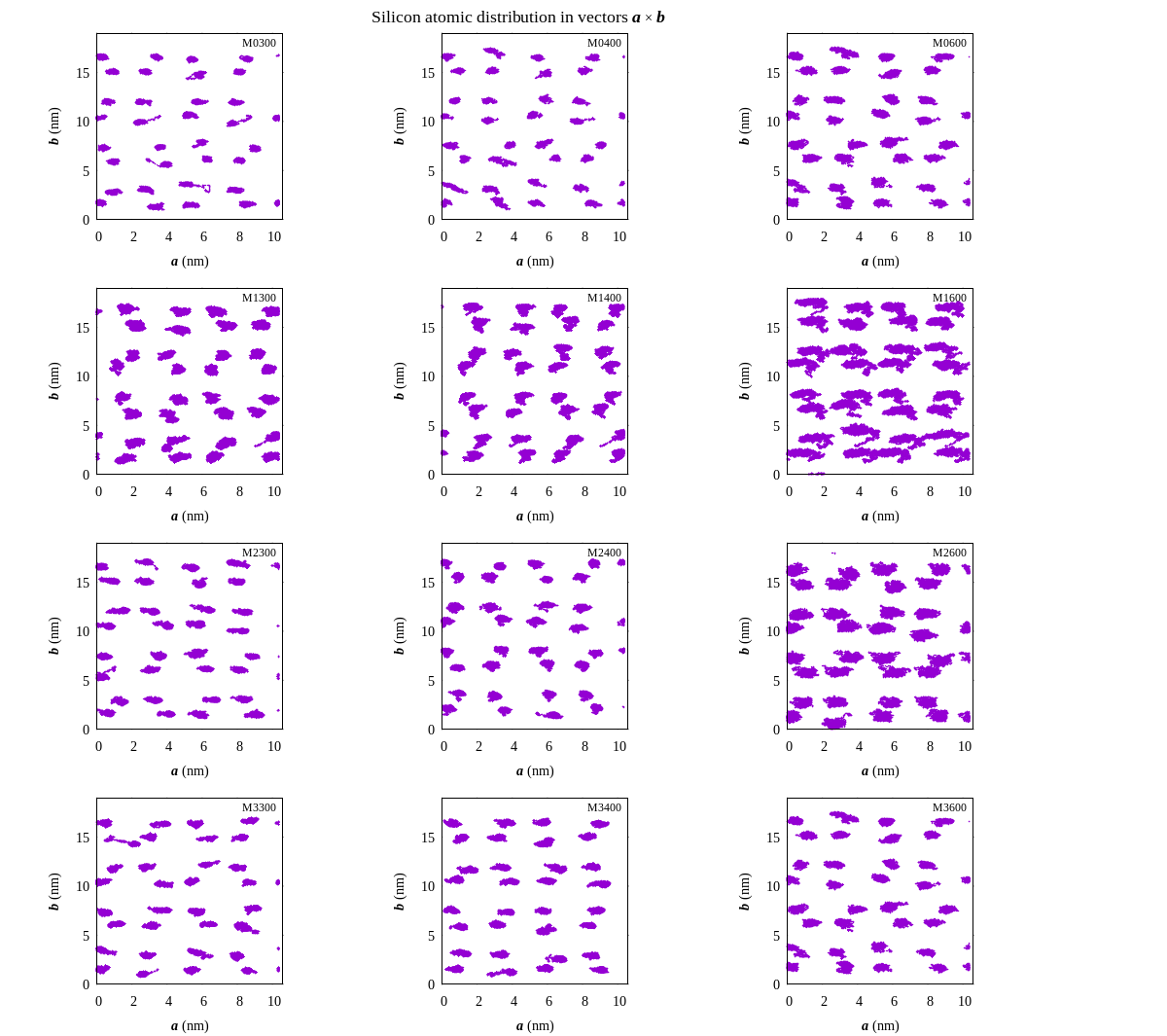


Figure S5: Mobility of silicon atoms represented by traces projected on the ***a****x****b*** plane.


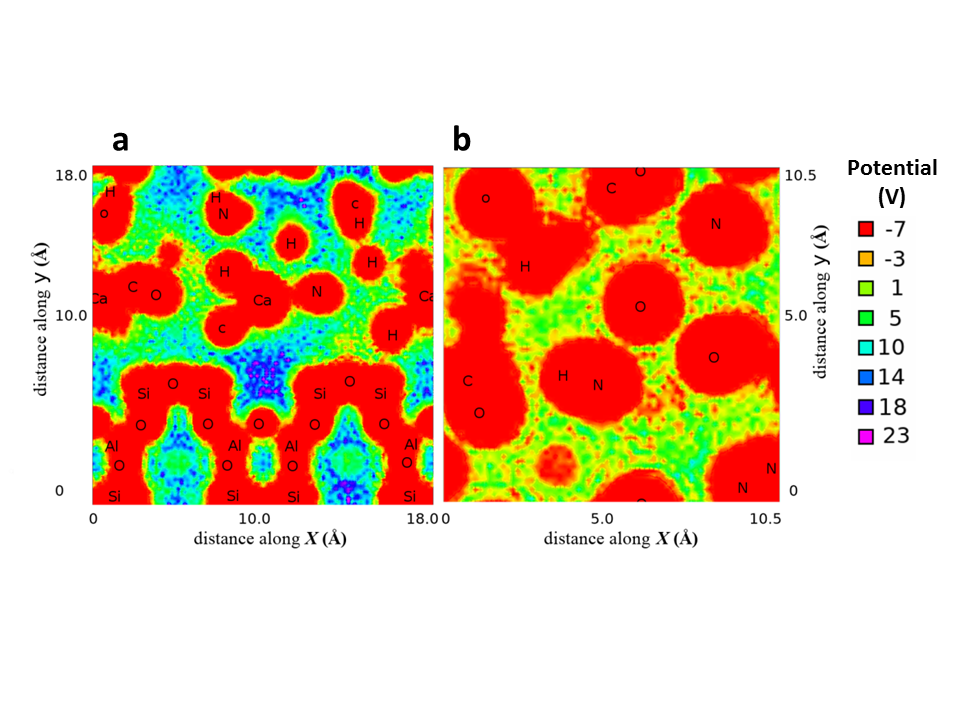


Figure S6: Model total electric potential (in V) calculated for selected frames from **m1** (a) and **g1** (b) CPMD trajectories, using PWsfc and pp.x modules of the Quantum Expresso code. The catalytic effect of the MT surface and Ca^+2^ is attributed to bigger gradient of V (i.e. stronger electric field) observed in (a) in comparison with (b). For details on PWscf and pp.x codes see https://www.quantum-espresso.org/.
